# Supplementary material for: Stable Isotope Analysis Reveals Habitat-Driven Dietary Niches of Lepus europaeus
Source: Animals (Basel). 2025 Dec 20;16(1):15. doi: 10.3390/ani16010015 (PMC12785083; doi:10.3390/ani16010015)
Supplement: Supplementary file 1 [file animals-16-00015-s001.zip › animals-4016255-supplementary.pdf]

animals

MDPI

Stable Isotope Analysis Reveals Habitat-Driven Dietary Niches of *Lepus europaeus*

Linas Balčiauskas [1,*], Rasa Vaitkevičiūtė-Koklevičienė [2,3], Andrius Garbaras [4], Jolanta Stankevičiūtė [2], Inga Garbarienė [4], and Laima Balčiauskienė [1]

Supplements

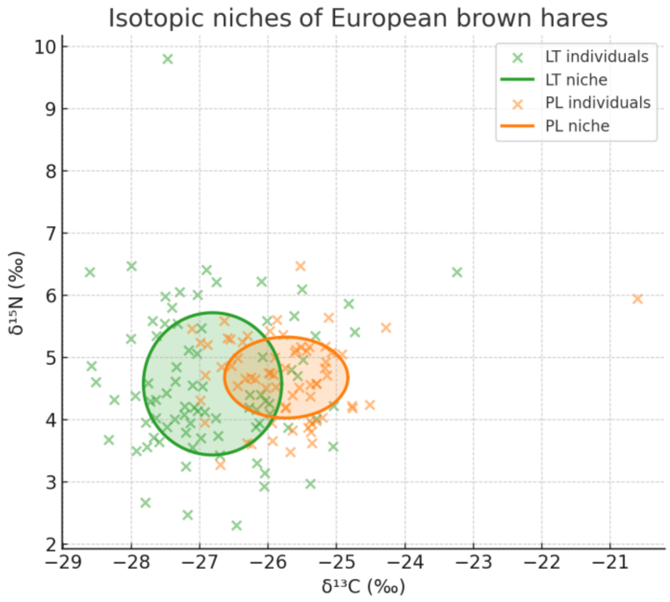

Figure S1. Central ellipses in isotopic space, representing fundamental niches of *Lepus europaeus* in Lithuania and Poland.

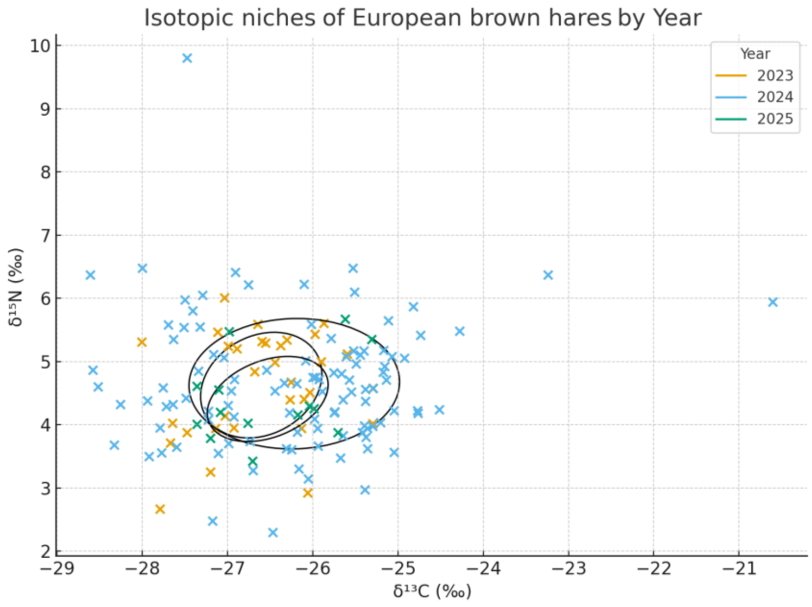

Figure S2. Central ellipses in isotopic space, representing fundamental niches of *Lepus europaeus* by year.

 https://doi.org/10.3390/ani16010015

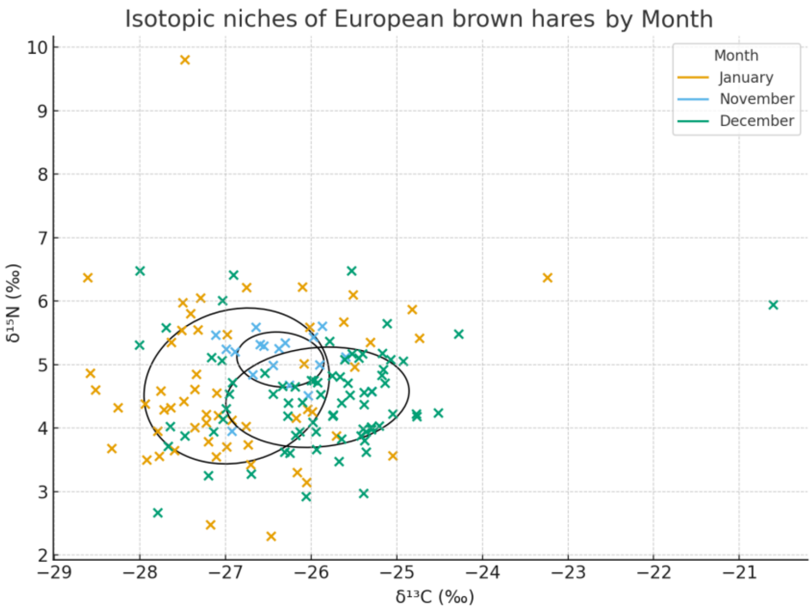

Figure S3. Central ellipses in isotopic space, representing fundamental niches of *Lepus europaeus* by month.

Table S1. Results of the General Linear Model for δ^{13}C and δ^{15}N values. Summary statistics include degrees of freedom (df), F-values, p-values, and effect sizes (partial η[2]) for each predictor. Statistically significant results ($p < 0.05$) are indicated in bold.

**Predictor**	**df**	**δ^{13}C**			**δ^{15}N**		
		F	p-value	Partial η[2]	F	p-value	Partial η[2]
Age	3	0.04	0.988	0.001	0.33	0.801	0.008
Country	1	6.11	0.015	**0.049**	3.62	0.060	0.030
Habitat group	5	3.20	0.010	**0.118**	3.24	0.009	**0.120**
Year	1	11.78	0.001	**0.090**	1.15	0.285	0.010
Month	1	3.37	0.069	0.028	6.82	0.010	**0.054**

Table S2. Isotopic niche metrics (SEA, SEAc, SEAb) based on δ^{13}C and δ^{15}N values in the hair of *Lepus europaeus from* Lithuania and Poland.

**Country**	**n**	**SEA**	**SEAc**	**SEAb_mean**	**SEAb_CI_low**	**SEAb_CI_high**
Lithuania	83	3.708352	3.754134	3.661958	2.924954	4.496121
Poland	68	1.866737	1.895021	1.836039	1.430996	2.307411

Table S3. Central positions (mean ± SD) and ranges of stable isotope ratios in the hair of *Lepus europaeus* in Lithuania and Poland depending on gender and age.

**Country**	**Gender/age**	**n**	**Mean δ^{13}C (‰) ± SD**	**Range (min–max)**	**Mean δ^{15}N (‰) ± SD**	**Range (min–max)**
Lithuania	Female	34	−26.85 ± 1.13	−28.52–−23.24	4.61 ± 1.37	2.30–9.80
	Male	31	−26.78 ± 0.85	−27.94–−25.05	4.45 ± 0.81	3.30–6.22
Lithuania	< 1 year	39	−26.91 ± 0.92	−28.58–−24.73	4.75 ± 1.26	2.30–9.80
	1–2 years	9	−26.90 ± 1.01	−28.25 –−25.05	4.15 ± 1.09	2.67–6.01
	2–3 years	16	−26.85 ± 0.80	−27.79 –−25.51	4.65 ± 1.11	2.48–6.41
	> 3 years	15	−26.64 ± 1.43	−28.61–−23.24	4.49 ± 1.00	2.97–6.37
Poland	< 1 year	42	−25.67 ± 0.64	−27.11–−24.28	4.71 ± 0.62	3.60–6.48
	1–2 years	6	−25.85 ± 0.69	−26.55 –−25.11	4.93 ± 0.62	4.03–5.64
	2–3 years	13	−25.69 ± 1.60	−27.00–−20.60	4.72 ± 0.71	3.48–5.95
	> 3 years	3	−26.03 ± 0.79	−26.69–−25.15	4.82 ± 0.13	4.68–4.93
